# Principles of brain plasticity in improving sensorimotor function of the knee and leg in patients with anterior cruciate ligament injury: a double-blind randomized exploratory trial

**DOI:** 10.1186/1471-2474-13-68

**Published:** 2012-05-10

**Authors:** Eva Ageberg, Anders Björkman, Birgitta Rosén, Ewa M Roos

**Affiliations:** 1 Department of Orthopedics, Clinical Sciences Lund, Lund University, Lund, Sweden; 2 Department of Health Sciences, Lund University, PO Box 157,, SE-221 00, Lund, Sweden; 3 Department of Hand Surgery, Clinical Sciences Malmö, Lund University, Lund, Sweden; 4 Research Unit for Musculoskeletal Function and Physiotherapy, Institute of Sports Science and Clinical Biomechanics, University of Southern Denmark, Odense, Denmark

## Abstract

**Background:**

Severe traumatic knee injury, including injury to the anterior cruciate ligament (ACL), leads to impaired sensorimotor function. Although improvements are achieved by training, impairment often persists. Because good sensorimotor function is associated with better patient-reported function and a potential lower risk of future joint problems, more effective treatment is warranted. Temporary cutaneous anesthesia of adjacent body parts was successfully used on the hand and foot to improve sensorimotor function. The aim of this study was to test whether this principle of brain plasticity could be used on the knee. The hypothesis was that temporary anesthesia of the skin area above and below the knee would improve sensorimotor function of the ipsilateral knee and leg in subjects with ACL injury.

**Methods:**

In this double-blind exploratory study, 39 subjects with ACL injury (mean age 24 years, SD 5.2, 49% women, mean 52 weeks after injury or reconstruction) and self-reported functional limitations and lack of trust in the knee were randomized to temporary local cutaneous application of anesthetic (EMLA®) (n = 20) or placebo cream (n = 19). Fifty grams of EMLA®, or placebo, was applied on the leg 10 cm above and 10 cm below the center of patella, leaving the area around the knee without cream. Measures of sensory function (perception of touch, vibration sense, knee kinesthesia) and motor function (knee muscle strength, hop test) were assessed before and after 90 minutes of treatment with EMLA® or placebo. The paired t-test was used for comparisons within groups and analysis of variance between groups, except for ordinal data where the Wilcoxon signed rank test, or Mann–Whitney test, was used. The number of subjects needed was determined by an a priori sample size calculation.

**Results:**

No statistically significant or clinically relevant differences were seen over time (before vs. after) in the measures of sensory or motor functions in the EMLA® group or in the placebo group. There were no differences between the groups due to treatment effect (EMLA® vs. placebo).

**Conclusions:**

Temporary cutaneous anesthesia of adjacent body parts had no effect in improving sensorimotor function of the knee and leg in subjects with severe traumatic knee ligament injury.

## Background

Severe traumatic knee injury, including injury to the anterior cruciate ligament (ACL), leads to impaired sensorimotor function. This is observed as, for example, proprioceptive deficiency, and reduced muscle strength and functional performance [1]. Although improvements are achieved by training interventions [2-4], impairment often persists despite such treatment [5]. Also, the achieved improvements are evident in measures of motor function (muscle strength, functional performance), but the possible influence of training on sensory function (proprioceptive acuity) remains uncertain [3,4,6]. The importance of sensorimotor function is reflected by its association with the patient’s perceived knee-related function and quality of life [7-9], and its potential protective role for detrimental long-term consequences, such as osteoarthritis (OA) [10-12]. From this perspective, treatment resulting in improved sensorimotor function would be of value for patients with knee injury and OA in the short and long term.

The primary motor (M1) and sensory (S1) cortex is organized somatotopically, where different body parts project to different parts of the M1 and S1. The somatotopic map does not represent the body in its actual proportions [13,14]. Instead, larger cortical areas are being assigned to sensitive parts or parts with complex motor demands such as the hands and face [15,16]. It is well known from animal and human experiments that temporary cutaneous anesthesia of one body part leads to cortical re-organization resulting in a corresponding silent area in the sensory cortex. This allows adjacent nearby body parts in the primary somatosensory cortex to rapidly expand at the expense of the silent cortical area [17,18]. This phenomenon, i.e., the manner in which the nervous system can modify its organization and ultimately its function [19,20], is often referred to as brain plasticity [21,22].

The ability of the central nervous systems (CNS) to change can also be used for therapeutic purposes, i.e., targeted plasticity [23] where weakened or lost functions can be strengthened. In healthy persons as well as in patients with median or ulnar nerve injuries, cutaneous anesthesia of the forearm has been shown to rapidly improve sensory function in the hand. The principle of temporary cutaneous anesthesia of adjacent body parts in combination with training was more effective in improving sensory function of the hand than training only [24,25]. The rapid improvement of sensory function and the enhanced effects 4 weeks after the last local anesthesia treatment [24,26], indicates that this intervention is clinically useful and relevant. Hypothetically, this principle could also be used to improve sensorimotor function of the knee. The ACL-injured knee may constitute a suitable model for this approach, because this peripheral musculoskeletal injury may also be regarded as a neurophysiological dysfunction [27,28]. An advantage is that the selective cutaneous anesthesia does not affect motor function of the leg, which means that the individual can use the leg during training while the skin is anesthetized.

In this study, we hypothesized that temporary anesthesia of the skin area above and below the knee would improve sensorimotor function of the ipsilateral knee and leg in subjects with severe traumatic knee ligament injury and self-reported functional limitations.

## Methods

### Subjects and randomization

Thirty-nine (19 women) subjects with ACL injury were included in this exploratory double-blind RCT. Inclusion criteria were: i) 18 to 35 years, ii) ongoing post-injury/post-surgery training, iii) ≥ 10 weeks after ACL injury, or ≥ 16 weeks after ACL reconstruction, iv) ability to perform a single-limb hop, v) self-reported functional limitations. Functional limitations were determined as knee-related problems in physical function and/or quality of life in at least 2 of 4 questions in the Knee injury and Osteoarthritis Outcome Score (KOOS) as follows: 1) at least moderate difficulty in jumping (subscale sport and recreation function, question 3); 2) at least moderate difficulty in twisting/pivoting on the injured knee (subscale sport and recreation function, question 4); 3) at least moderate trouble with lack of confidence in the knee (subscale quality of life, question 3); 4) at least moderate difficulty with the knee in general (subscale quality of life, question 4). Exclusion criteria were a history of other major orthopedic lesions, such as previous knee injury or fracture, and allergic reactions to anesthetic agents. The patients were enrolled at a sports physical therapy clinic by the test leader. They all had neuromuscular training [1,29], supervised by either of eight physical therapists at this clinic. Subject characteristics, including activity level [30] and self-reported outcomes assessed by the Knee injury and Osteoarthritis Outcome Score (KOOS) [31,32], are given in Table 1.

**Table 1:** Characteristics of the subjects

**Characteristic**		**EMLA group (n = 20)**	**Placebo group (n = 19)**
Age (y)*		25 (5.6)	23 (4.9)
Women (n)		10	9
BMI*		23.7 (3.1)	24.3 (2.9)
ACL injury (ACL reconstruction) (n)		6 (14)	9 (10)
Time after ACL injury or ACL reconstruction (weeks)^‡^		54 (17–315)	48 (10–243)
Previous contralateral ACL injury/reconstruction (n)		2	2
Tegner activity level^†^		2 (2–4)	4 (2–6)
KOOS subscales*			
Pain		82 (10.8)	78 (12.2)
Symptoms		74 (12.0)	72 (14.3)
ADL		90 (8.6)	90 (10.4)
Sport/Rec		47 (20.9)	47 (24.7)
QOL		41 (12.4)	40 (10.5)

* Mean (SD), † median (quartiles), ^‡^ mean (range), BMI; body mass index.

ACL reconstruction: hamstring tendons graft, n = 23, patellar tendon graft, n = 1.

The Tegner Activity Scale, ranges from 0 to 10, least to hardest strenuous activity for the knee. A Tegner activity level of 2 is equal to recreational sports such as golf, cycling and swimming, an activity level of 4 is equal to recreational sports such as jogging, aerobics, or cross-country skiing and a Tegner activity level of 6 is equal to recreational sports such as tennis or badminton and competitive sports such as orienteering.

The subjects were randomly allocated, using a random number generator, to temporary anesthesia using a local anesthetic cream, EMLA®, (EMLA® group) or a placebo cream (oil and water emulsion) (placebo group). To ensure an equal number of men and women in each group two computer-generated randomization lists, one for women and one for men, were drawn up by a biostatistician and given to the assessor. The assessor allocated the next available number on entry into the trial, assigning the subjects to treatment/placebo. The Research Ethics committee of Lund University approved the study (LU 107/2007), and all subjects gave their written informed consent.

### Protocol and masking

**Figure 1:**
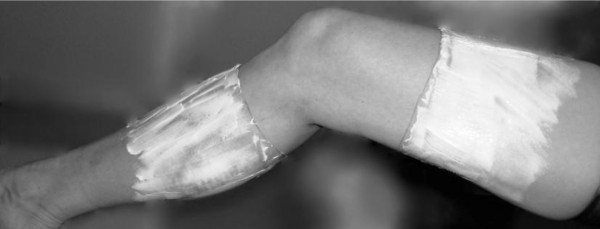
**Application of local anesthetic or placebo cream.** EMLA, or placebo, applied on the leg 10 cm above and 10 cm below the center of patella, leaving the area around the knee without cream.

Twenty subjects received a local anesthetic cream containing 2.5% lidocaine and 2.5% prilocaine (EMLA®, AstraZeneca, Södertälje, Sweden) and 19 subjects received a placebo cream of an oil and water emulsion (DAX, Opus Health Care Inc., Malmö, Sweden). The two creams were identical in color, consistency and packaging. A staff member, not participating as an assessor or subject in the study, distributed the packages with cream to the assessor. Fifty grams of EMLA®, or placebo [33], was applied circumferentially on the leg 10 cm above and 10 cm below the center of patella, leaving the area around the knee without cream (Figure 1) [34]. The skin areas where the EMLA®/placebo was applied were covered with film wrap and a Tubigrip® stocking (MEDLOCK Medical, Oldham, UK). After 90 minutes, during which time the subject was seated, the EMLA®/placebo was carefully washed off. The test leader and the subjects were blinded to group allocation, and the subjects were told not to reveal any possible anesthetic sensation. Therefore, the presence or absence of anesthesia in the area where the EMLA®/placebo was applied was not verified by the assessor or the subject. The success of blinding related to cream was not evaluated.

### Outcome measures

Measures of sensory and motor functions were assessed before and after 90 minutes of treatment with EMLA® or placebo according to the protocol of our previous study [34]. The tests were performed in the order that they are described below. EMLA®/placebo was applied and all tests were performed on the injured leg only (in those four with a previous contralateral knee injury, the recently injured leg was tested). An experienced assessor, who received explicit guidelines and thorough training and pilot-testing prior to study start, performed the measurements.

#### Measures of sensory function

Three measures of sensory function were used; perception of touch, vibration sense and knee kinesthesia. Lower values in these tests indicate better sensory function.

##### Perception of touch

Semmes-Weinstein monofilaments (SWM) were used for assessing perception of touch at the most prominent point of the medial and lateral femoral condyles, just proximal of the joint space (areas where no cream was applied). In our previous study, one site (the medial femoral condyle) was assessed only [34]. Because a ceiling effect was found in that study [34], two sites were included in the current study. Prior to the test, the SWM (nr 4.31, 2.0 g) was demonstrated on the patient’s styloid process of the hand, so that the subjects could familiarize themselves with the test. Thereafter, the subjects lay in a supine position and were asked to close their eyes, concentrate on their knee and respond when they felt any sensation of touch. The assessment was performed according to a standardized procedure [35]. Each monofilament, starting with the thinnest and continuing with thicker until response to sensation, was applied perpendicular to the skin for 1.5 seconds and lifted for 1.5 seconds. The filament was applied 3 times to the same spot and was bent each time to exert the specific pressure. Feeling the monofilament was recorded when at least one out of three applications was identified by the patient [35].

##### Vibratory perception threshold

Vibratory perception threshold (VPT) was assessed by a biothesiometer (Bio-Medical Instrument, Newbury, OH, USA), as described [34]. Prior to the test, the Biothesiometer was demonstrated on the patient’s ulna styloid process, so that the subjects could familiarize themselves with the test. Thereafter, the subjects lay in a supine position and were asked to close their eyes, concentrate on their foot/knee and respond when they felt any sensation of vibration. The biothesiometer tip was held with uniform pressure at two sites: the most prominent point of the medial malleolus and the medial femoral condyle (same location as that for testing perception of touch). Three consecutive measurements were taken on each site, and the amplitude was replaced to zero between each measurement without moving the biothesiometer tip from the location. The amplitude was increased by 1 Volt per second until the subjects responded to a sensation of vibration. This was noted as the VPT. The first measurement was regarded a trial test, and was, thus, excluded from the analysis. If the difference between the second and third measurement was more than 20%, 2 additional tests were taken. The mean of the second and third, or fourth and fifth, measurements was used in the analysis. High reliability has been reported for the biothesiometer [36,37]. Vibration data for the patients and controls was reported previously [38].

##### Knee kinesthesia

Kinesthesia was measured in a specifically designed apparatus, which has been used and described in detail in previous studies [39,40]. The subjects lay in a lateral decubitus position, were asked to close their eyes, concentrate on their knee and respond when they felt any sensation of movement in their knee. Measurements of the threshold for detection of passive motion (TDPM) were performed towards knee extension (TE) and knee flexion (TF) from the starting position of 20° knee joint flexion, giving the variables TE20 and TF20. The median values of three consecutive measurements of these two variables were determined. The variables from the 20° starting position (TE20 and TF20) have been found to be reliable in uninjured subjects [41]. The sum of TE20 and TF20, giving an index value, was used for statistical analysis.

#### Measures of motor function

Two measures of motor function were used; the one-leg hop test for distance and knee extensor muscle power. Higher values in these tests indicate better motor function.

##### One-leg hop test for distance

The one-leg hop test for distance with the arms free, aiming at a more functional execution of the hop, was used. The one-leg hop test is widely used for predicting functional knee stability [1,42]. Muscle strength, balance and confidence in the knee are contributing factors to the performance of this test. The subjects were told to hop as far as possible, taking off and landing on the same foot, maintaining their balance for about 2–3 seconds. The test was performed three times, the hop distance being measured (in cm) from toe in the starting position to heel in the landing position. If the subject improved more than 10 cm between the second and third hop, additional hops were performed until an increase of less than 10 cm was measured. A trial one-leg hop preceded the measurements. The subjects wore shoes, e.g., sneakers. The mean value of the three best hops was used in the analysis. The reliability of this test is high in subjects with ACL injury [43].

##### Knee extension power

The muscle strength test was performed as described, with standardized verbal instructions and encouragement [5,44]. The subject was seated, using an individual seating position, in a knee extension weight training machine (Precor, Icarian, Borås, Sweden). The test was performed with the injured leg, and the other leg was fixed in place using a strap. Before the strength test, the subjects completed ten repetitions at a sub-maximum weight, followed by five repetitions using a somewhat higher sub-maximum weight. The subject then performed a single repetition, of approximately 90% of their maximum, to select the appropriate starting weight.

On a given signal, the subject was asked to extend his/her knee as quickly and forcefully as possible from approximately 110^o^ of knee flexion to full knee extension (0^o^ of flexion). The distance the weight stack was lifted and the time it took to fully extend the knee was measured with a linear encoder connected to the weight stack of the machine. In all, the subjects performed five maximum trials at five weight levels, until a decrease in power was seen. The weight was increased by 5 kg for each trial. The rest period between trials was 30 s, which was considered to be sufficient for full recovery. The average power was calculated by Muscle Lab, a computerised muscle function measuring system (Ergotest Technology, Oslo, Norway). If a subject reported pain in the knee during a test, which occurred in a few cases, this particular test was cancelled and the measurement was repeated. Data was missing for 2 patients (1 patient did not perform the test because of knee pain, and too few accurate values to calculate average power for 1 patient). Average power (W) was used in the analysis.

### Statistical analysis

The number of subjects needed was determined by an a priori sample size calculation from our previous study [34]. No primary outcome measure was determined, since the study has an exploratory character. We expected to find an improvement in more than one of the variables to interpret the results as an effect from treatment. For knee kinesthesia, sample size calculations revealed that at least 12 subjects were needed to detect an improvement by treatment of 30% within groups (SD_diff_ 0.49), with 80% power at the 5% significance level. For vibration sense, 13 subjects were needed to detect an improvement of 20% (SD_diff_ 3.3) within groups. For the one-leg hop test, and knee extension power, less than 10 subjects were needed to detect an improvement by treatment of 10% within groups, with 80% power at the 5% significance level. Based on these sample-size calculations, we included 40 subjects. The paired t-test was used for comparisons within groups and analysis of variance, adjusting the variables for activity level, between groups. All variables had Shapiro-Wilk statistic of >0.90, except knee kinesthesia and VPT at the medial malleolus. Because some variables were not normally distributed, the results were confirmed using non-parametric statistics. Wilcoxon signed rank test, or Mann–Whitney test, was used for ordinal data (perception of touch). Effect size was calculated by taking the difference between the means before and after EMLA®/placebo and dividing it by the SD of the same measure before EMLA®/placebo [45]. An effect size of <0.50 was considered small, 0.50 to 0.79 moderate, and ≥0.80 large [45]. A level of *p* ≤ 0.05 was chosen to indicate statistical significance. Group allocation was concealed to the person analyzing the data, until the results were completed.

## Results

There were no differences between the groups due to treatment effect (EMLA® vs. placebo) (Table 2). No statistically significant or clinically relevant differences were seen over time (before vs. after) in the measures of sensory or motor functions in the EMLA® group or in the placebo group.

**Table 2:** Results for outcomes of sensory and motor functions in the EMLA and placebo groups

	**EMLA group (n = 20)**			**Placebo group (n = 19)**			**EMLA vs. placebo**
	**Before**	**After**		**Before**	**After**		
	**Mean (SE)**	**Mean (SE)**	**Mean diff (95% CI)**(after minus before)	**Mean (SE)**	**Mean (SE)**	**Mean diff (95% CI)**(after minus before)	**Mean diff (95% CI)**(EMLA minus placebo)
**Sensory function**							
SWM med fem cond (grams)†*	0.02 (0.008:0.02)	0.008 (0.008:0.02)	*p* = 0.430	0.008 (0.008:0.02)	0.008 (0.008:0.04)	*p* = 0.134	*p* = 0.276
SWM lat fem cond (grams)†*	0.008 (0.008:0.02)	0.008 (0.008:0.02)	*p* = 0.442	0.008 (0.008:0.04)	0.008 (0.008:0.03)	*p* = 0.505	*p* = 0.951
VPT med mall (Volt)	9.2(0.6)	10.3(0.8)	1.1(0.4:1.8)	9.7(0.60)	10.2(0.68)	0.5(−0.1:1.1)	0.6(−0.4:1.5)
VPT med fem cond (Volt)	15.5(1.27)	15.1(1.51)	−0.4(−1.9:1.1)	16.0(1.20)	15.5(1.22)	−0.6(−2.4:1.3)	0.0(−2.4:2.4)
TDPM (degrees)	2.69(0.33)	2.52(0.27)	−0.16(−0.73:0.41)	2.74(0.37)	2.84(0.54)	0.11(−1.0:1.27)	−0.28(−1.57:1.01)
**Motor function**							
One-leg hop (cm)	79.2(12.4)	83.3(7.61)	4.0(−0.1:8.2)	90.6(9.9)	100.0(9.7)	9.5(3.6:15.3)	−6.4(−13.4:0.6)
Knee ext power (W)*	130.0(12.4)	139.5(14.0)	9.5(1.4:17.7)	156.9(15.4)	158.3(15.5)	1.5(−8.1:11.0)	6.9(−5.5:19.4)

*SWM* Semmes-Weinstein monofilaments, *VPT* vibration perception threshold, *Med mall* medial malleolus, *Med fem cond* medial femoral condyle, *Lat fem cond* lateral femoral condyle, *TDPM* threshold for detection of passive motion, *ext* extension.

† median (quartiles).

* Missing data 1 patient for SWM (placebo group) and 2 patients for knee ext power (1 patient in each group).

Mean and standard error (SE) and mean difference (95% CI) (after minus before) for the tests of sensory function (perception of touch, vibration sense, kinesthesia) and motor function (one-leg hop test, knee extension power) before and after treatment with EMLA/placebo, and mean difference (95% CI) (EMLA minus placebo) between the EMLA and placebo groups (t-test). Median (quartiles) and p-value (Wilcoxon singed rank test, Mann–Whitney test) given for perception of touch (ordinal data).

### **Sensory function before and after treatment with EMLA**® **or placebo**

There were no differences between the groups in effects of treatment for the measures of sensory function (Table 2). The effect sizes were generally small in the EMLA® group (between 0.08 and 0.44) and in the placebo group (between 0.07 and 0.19, except for perception of touch at the medial femoral condyle; effect size 0.78). No differences were found before vs. after treatment for perception of touch, vibration sense at the medial femoral condyle, or TDPM in the EMLA® group. A higher VPT at the medial malleolus, indicating poorer vibration sense, was found after compared with before treatment. No differences were found between assessments (before vs. after) for perception of touch, vibration sense, or kinesthesia in the placebo group (Table 2).

### Motor function before and after treatment with EMLA® or placebo

There were no differences between the groups in effects of treatment for the measures of motor function (Table 2). The effect sizes were small in the EMLA® group (one-leg hop test 0.11, knee extension power 0.18) and in the placebo group (one-leg hop test 0.22, knee extension power 0.02). The patients in the EMLA® group had higher knee extension power, and the patients in the placebo group jumped a longer distance, after compared with before treatment (Table 2).

## Discussion

The hypothesis of this exploratory RCT on principles of brain plasticity in improving sensorimotor function of the knee in subjects with severe traumatic knee ligament injury was not confirmed. No effect was found of temporary cutaneous anesthesia of the skin area above and below the knee on sensorimotor function of the ipsilateral knee and leg in these subjects.

The forearm is located next to the hand in the somatotopic map [13,14] and by anaesthetizing the forearm, the cortical hand area can expand over the forearm area [46]. Thus, more nerve cells can be available for the hand, resulting in improved hand function. This principle has been successfully used in subjects with or without hand nerve injury [24,25,47], and was recently also efficiently applied to the foot in subjects with or without diabetes [33,48].

Diminished activation in several sensorimotor cortical areas has been observed in subjects with ACL injury compared with controls, indicating that the injury causes re-organization of the central nervous system [28]. Conversely, it may be assumed that cortical re-organization can be achieved by an efficient intervention. By assessing sensory function in the knee, we indirectly assessed a possible cortical re-organization. An improved sensory function, as found in the hands following forearm anesthesia [46], would indicate a cortical re-organization with an expanded cortical knee area in subjects with anesthetized leg areas. Although no such changes were seen, this does not completely rule out cortical changes following treatment. However, if cortical changes did occur they were likely very subtle. This, in combination with the fact that the knee normally has a very small representation in the somatosensory cortex, makes is difficult or perhaps even impossible in this case to detect changes in the cortical knee area using neuroimaging methods such as fMRI. With this in mind, and because no effects were found in the sensorimotor function tests in the present study or in a previous study in healthy subjects [34], we decided not to perform fMRI because the likelihood of finding an cortical expansion-difference would be small.

Neurophysiologic mechanisms in the lower extremity may also differ from those in the upper extremity. Large overlaps in the sensorimotor activation have been shown following movement of the knee, ankle and toes as opposed to the fingers [27]. However, the same plasticity mechanisms likely occur in both the upper and lower extremity, thus making it possible to manipulate plasticity mechanisms also in the lower extremity in order to improve sensorimotor function, such as that reported for the foot [33,48].

Because this was an RCT with a blinded design, that is, both the assessor and the subject were blinded to group allocation, numbness in the area where the EMLA®/placebo was applied (see Figure 1) was not verified. However, the amount of EMLA® used (50 g) is a dosage of active substance per cm^2^ well within the recommendations to achieve cutaneous anesthesia.

The placing of the cream (above and below the knee) is most likely adequate in order to expect an increased cortical knee representation. Because the cortical area devoted to the lower extremity is small compared to the hand, we expected that a larger deafferented skin area was needed (compared to the upper extremity) in order to allow the knee to expand in the primary somatosensory and motor cortex. Therefore, the EMLA® or placebo was applied circumferentially on the skin both above and below the knee.

The effect sizes were generally small for all outcome measures, indicating that the magnitude of change by treatment was small. In previous studies on the hand and foot, focus was on assessing improvements of temporary anesthesia in sensory function (perception of touch, vibration sense) [24,25,33,47,48], as these measures are relevant to the patient’s daily activities [24]. Because we applied this concept to the knee, these sensory measures were included also in the present study. The perception of touch of the knee is not as delicate, discriminative or vital as in the hand or the foot sole. Therefore, the relevance of this measure for patients with knee injury, as used in the present study, may be questioned. The first study evaluating vibration sense in patients with ACL injury was recently reported [38]. Vibration perception threshold was not impaired in these patients compared with matched controls [38]. Perhaps a ceiling effect was present, limiting the chance of improving perception of touch and/or vibration sense in these patients by intervention using temporary anesthesia.

Several studies have shown a deficiency in proprioceptive acuity (kinesthesia, joint position sense) in patients with ACL injury; recently reported in a review [49]. While measures of motor function (muscle strength, functional performance) appear relevant for patients with knee injury [7-9], the association between sensory function (proprioceptive acuity) and patient-reported and motor functions is generally low [49]. For this reason, the development of more accurate and precise methods for assessing sensory function was proposed [49]. Possibly, measures of motor function may be more essential for activities of daily life and more demanding activities than measures of sensory function for subjects with knee injury. However, the possibilities of affecting motor outcomes by temporary anesthesia may be more difficult to achieve than for sensory outcomes [24].

The major strength of the current and previous [34] studies is the design; the subjects were randomized to anesthetic or placebo cream, the test leader was blinded to group allocation, and group allocation was concealed to the person analyzing the data until the results were completed. However, from the results of the present and previous [34] studies, temporary cutaneous anesthesia was not a successful intervention to improve sensorimotor function for patients with severe traumatic knee ligament injury. The need for development and evaluation of methods to improve the effects of treatment on sensorimotor function after knee injury and knee OA remains.

## Conclusions

The principle of temporary cutaneous anesthesia of adjacent body parts had no effect in improving sensorimotor function of the knee and leg in subjects with severe traumatic knee ligament injury.

## Competing interests

The authors declare that they have no competing interests.

## Authors’ contributions

EA contributed to the design of the study, was responsible for acquisition, analysis and interpretation of data, and drafted the manuscript. AB and BR contributed to the design of the study and critically revised the manuscript. ER contributed to the design of the study, participated in analysis and interpretation of data, and critically revised the manuscript. All authors read and approved the final version.

## Pre-publication history

The pre-publication history for this paper can be accessed here:

http://www.biomedcentral.com/1471-2474/13/68/prepub
